# A Study of Theoretical Analysis and Modelling of Microalgal Membrane Photobioreactors for Microalgal Biomass Production and Nutrient Removal

**DOI:** 10.3390/membranes14120245

**Published:** 2024-11-22

**Authors:** Yichen Liao, Pedram Fatehi, Baoqiang Liao

**Affiliations:** Department of Chemical Engineering, Lakehead University, 955 Oliver Road, Thunder Bay, ON P7B 5E1, Canada; yliao1@lakeheadu.ca (Y.L.); pfatehi@lakeheadu.ca (P.F.)

**Keywords:** microalgae, photobioreactor, mathematical simulation, biological performance, wastewater treatment

## Abstract

This study presents a theoretical and mathematical analysis and modelling of the emerging microalgal membrane photobioreactors (M-MPBRs) for wastewater treatment. A set of mathematical models was developed to predict the biological performances of M-MPBRs. The model takes into account the effects of hydraulic retention time (HRT), solid retention time (SRT), and the N/P ratio of influent on the biological performance of M-MPBRs, such as microalgal biomass production and nutrient (N and P) removals. The model was calibrated and validated using experimental data from the literature. This modelling study explained that prolonged SRT could promote biomass production and nutrient removal, while prolonging HRT exhibited a negative effect. Furthermore, biomass production could be improved by augmenting nutrient loading, and nutrient removal would be limited under insufficient conditions. The modelling results demonstrated that the best performance was achieved at HRT = 1 d and SRT = 40 d for typical municipal wastewater with an influent N concentration = 40 mg/L. The modelling results are in good agreement with the experimental results from the literature. The findings suggest that the proposed models can be used as a powerful mathematical tool to optimize these parameters to improve the removal of nutrients (N and P), as well as the productivity of biomass in M-MPBRs. This study provides new insights into the use of mathematical models for the optimal design and operation of the emerging M-MPBRs for sustainable wastewater treatment.

## 1. Introduction

Microalgae are photo-autotrophic microorganisms that can consume nutrients such as nitrogen and phosphorus (N and P) for growth in an aqueous system. Microalgae photobioreactors have been studied for decades, and microalgal biomass has been utilized in a wide range of areas, such as nutrients, cosmetics, and pigment applications [1,2,3]. As a traditional biological process for wastewater treatment, activated sludge is facing challenges, such as the production of large amounts of harmful sludge and the low efficiency of nutrient (N and P) removals [4,5,6]. Compared to the traditional activated sludge biological treatment, microalgae-based wastewater treatment could not only achieve effective nutrient removals but also produce a high-lipid content feedstock for downstream applications, such as biofuel or biodiesel production to alleviate the pressure of energy shortage [7,8,9]. Furthermore, Palandi and Taghavijeloudar found that the addition of iron and zinc in municipal wastewater could improve microalgal performances, such as biofuel/bio-product production and pollutant removal, indicating that a microalgae-based system has a high potential for dealing with heavy metal wastewater [10]. Microalgae cultivation has not been industrialized for wastewater treatment due to handicaps, such as the diluted suspended microalgae concentration, the difficulty in lipid extraction, and the high cost of microalgae harvesting and dewatering [11,12,13].

Compared to conventional photobioreactors (either open ponds or closed bioreactors), the concept that integrates membrane separation technology with a microalgae-based biological process guarantees a better effluent quality, concentrated microalgal biomass, and the decoupling of hydraulic retention time (HRT) and solid retention time (SRT) [14,15,16]. However, challenges such as membrane fouling and the high cost of maintenance and the short life cycle of the membrane are not avoidable by the application of membrane technology [14,15,16,17]. As mentioned in the literature, the adjustment of operating conditions of the M-MPBRs contributed to the mitigation of severe membrane fouling and the improvement of biological performance [18,19]. Even though the effects brought by operating parameters could be diverse, the adjustment of those parameters should focus on the improvements of pollutant removals (N and P removals) and microalgal biomass production for the purposes of wastewater treatment and biomass harvesting for biofuels and feedstocks for other value-added products. Zhao et al. [20] found that HRT was a key condition that affected biomass productivity and nutrient removal efficiency. Short HRTs facilitated microalgae growth due to the higher nutrient loading, while long HRTs contributed to a better nutrient removal efficiency but sometimes caused nutrient limitations [20]. Furthermore, Xu et al. [21] revealed that the *C. vulgaris* concentration increased from 895 mgCOD-equivalent/L to 1473 mg COD-equivalent/L, when the HRT of M-MPBR reduced from 24 h to 12 h. However, they also found that a higher HRT (24 h) achieved a higher removal efficiency of nitrogen (66%) and phosphorus (91%) [21]. Moreover, another key parameter affecting biomass concentration and pollutant removal is SRT. As indicated in the literature, the uncoupled HRT and SRT in the M-MPBR system could maintain a high concentration of biomass in the system (longer SRT) while dealing with a high nutrient loading (short HRT) [22]. Thus, this high concentration of biomass can result in faster and more stable nutrient removal. Honda et al. [23] found that the concentration of *Chlorella* increased with an increase in SRT from 9 d to 18 d. As essential nutrients for microalgal biomass production, N and P loadings are also important in affecting the M-MPBR performance, particularly the microalgal biomass production [14,15,16,17]. The higher nutrient loading could promote microalgae growth, but the growth decline might happen at an extremely higher level, due to the inhibition caused by excessive nutrients [24]. Some studies reported a similar observation that microalgal biomass concentration increased with the augment of nutrient concentrations in influent, but a dramatic drop occurred at an extremely high level of nutrients (both N and P) [25,26]. Among the various factors (SRT, HRT, nutrient loading, light intensity, temperature, and so on) that influence M-MPBR performance, SRT, HT, and nutrient loading are the most cost-effective and easiest way to be adjusted for optimal microalgae cultivation.

Many studies have been conducted to identify the optimal operating conditions to take full advantage of an M-MPBR for wastewater treatment and the following downstream product economic potential [27,28,29]. However, the optimal conditions could vary due to changes in the microalgae species, M-MPBR configuration, and industrial requirements. Thus, the optimization of operating conditions and wastewater characteristics is still insufficient, and it is time-consuming and expensive because it requires considerable amounts of laboratory experiments. The introduction of mathematical modelling to predict the MBPR performance opens a new door for the optimal design and operation of M-MPBRs and is thus suggested before the experiment work to save time and cost [30]. Mathematical models could simulate the specific microalgae activity in an M-MPBR by using biokinetic models and mass balances. Several mathematical models have been proposed to investigate the effects of different microalgae species on the biological performance of different types of conventional (non-membrane-based) photobioreactors [22,31,32]. However, there is no mathematical model and modelling work published for the emerging M-MPBR systems in the literature, although mathematical modelling has been widely used to study the biological performance of bacteria MBRs for COD removal [33,34].

The objective of this work was to investigate the influence of operating conditions (SRT and HRT) and wastewater characteristics (N, P concentration and N/P ratios) on the biological performance (N and P removals, and microalgal biomass production) of M-MPBRs by using mathematical models established based on the mass balance concept and microbial biokinetic models. The proposed mathematical models were calibrated and validated using experimental data from previous studies published in the literature. The mathematical models presented here are proven to be a powerful tool to optimize the design and operation of M-MPBRs.

## 2. Methods–Model Design and Model Variables

### 2.1. Model Design

**Figure 1:**
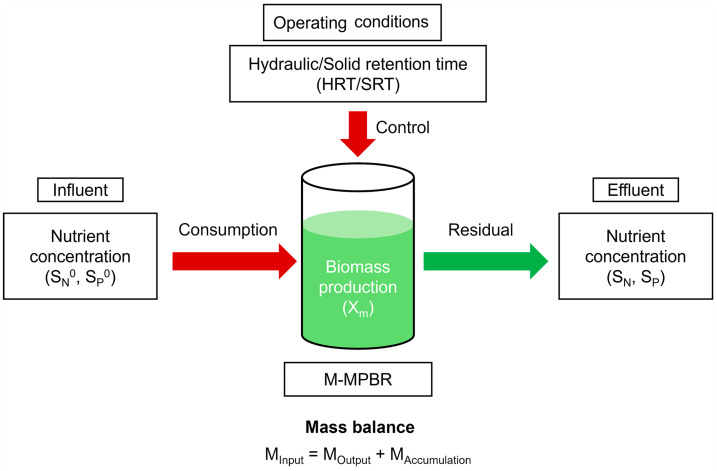
The model process development of the biological process profile involved in the M-MPBR system. (1.5 column).

The model development proceeded through the steps indicated in Figure 1. Mathematical models were based on the mass balance concept and microbial kinetic models. Biomass and nutrient (N and P) balances are in the form of ordinary differential equations for lumped systems. Solids and liquid phases’ mass balances were considered in the M-MPBRs, and the mass balance equations were derived, based on the following assumptions:

1. The model only predicts the biological performance of M-MPBRs under a steady state, which would not provide the details of performance development during the transit period.
2. The illumination and gas (CO_2_) supply are continuous and sufficient in the cultivation system of microalgae; thus, they are assumed not to be limiting factors of microalgae growth in the models.
3. Microalgae microbial biokinetic model follows the Monod equation for multi-nutrients (N and P) as the limiting factors.
4. The nutrient (N and P) consumption mechanism is dominated by microalgae uptake; thus, other nutrient removal mechanisms such as the nitrification interaction between the bacteria and microalgae are not taken into account, as the models are for microalgae-only MPBRs (no consideration of bacterial contamination or microalgal-bacterial consortia).

In general, HRT and SRT are decoupled as individual factors for the M-MPBR, which could be expressed by the following equations:

(1) HRT=V/Q

(2) $$\mathrm{SRT}=\frac{VX_{m}}{Q^{w}X_{m}}=V/Q^{W}$$

*V*—Effective volume of M-MPBR, L;

Q—Flow rate of influent, $L\cdot d^{-1}$;

$Q^{w}$—Waste rate of microalgae suspension, $L\cdot d^{-1}$;

$X_{m}$—Microalgal biomass concentration, $mg\cdot L^{-1}$.

In the M-MPBR, the accumulation of microalgal biomass is equal to the sum of all biomass changes, including the initial amount of inoculum, the increasing biomass caused by microalgal growth over time, and the deduction of the loss of cell death and daily discard biomass. Biomass mass balance can be expressed as the following equation:

(3) $$V\frac{dX_{m}}{dt}=QX_{m}^{0}-Q^{w}X_{m}+V(\frac{\mu_{m}X_{m}S_{N}S_{P}}{\left(K_{N}+S_{N}\right)\left(K_{P}+S_{P}\right)}-X_{m}k_{d-m})$$

$X_{m}^{0}$—initial concentration of microalgae in M-MPBR, $mg\cdot L^{-1}$gL^−1^;

$X_{m}$—microalgae concentration in discard suspension, $mg\cdot L^{-1}$gL^−1^;

$\mu_{m}$—maximum growth rate of microalgae, d^−1^;

$S_{N}$—total nitrogen concentration in M-MPBR and effluent, $mg\cdot L^{-1}$;

$S_{P}$—total phosphorus concentration in M-MPBR and effluent, $mg\cdot L^{-1}$;

$K_{N}$—half saturation constant of NH_4_^+^-N, mg N L^−1^;

$K_{P}$—half-saturation constant of HPO_4_^2−^-P, mg P L^−1^;

$k_{d-m}$—decay coefficient of microalgae, d^−1^.

Under the steady state, biomass maintains a relatively stable value, which means the changing rate $V\frac{dX_{m}}{dt}$ = 0, and the initial biomass $X_{m}^{0}$ is assumed as zero.

Thus, the equation can be simplified to the following:

(4) $$VX_{m}(\frac{\mu_{m}S_{N}S_{P}}{\left(K_{N}+S_{N}\right)\left(K_{P}+S_{P}\right)}-k_{d}){=Q}^{w}X_{m}$$

When Equation (4) is divided by $Q^{w}X_{m}$, then the following equation is obtained:

(5) $$\frac{V}{Q^{w}}(\frac{\mu_{m}X_{m}S_{N}S_{P}}{\left(K_{N}+S_{N}\right)\left(K_{P}+S_{P}\right)}-k_{d-m})=1$$

Based on Equation (2), Equation (5) can be further simplified:

(6) $$SRT(\frac{\mu_{m}S_{N}S_{P}}{\left(K_{N}+S_{N}\right)\left(K_{P}+S_{P}\right)}-k_{d-m})=1$$

Moreover, the accumulation of nutrients in the M-MPBR is equal to the sum of the initial medium concentration ($QS_{i}^{0}$), the deduction of nutrients in the effluent ($QS_{i}$), and the consumption of microalgal metabolism ($\gamma_{{\omega t}_{i}}$). Thus, the nutrient (*N* and *P*) mass balance can be expressed as the following equation:

(7) $$V\frac{dS_{i}}{dt}=QS_{i}^{0}-QS_{i}+\gamma_{{\omega t}_{i}}$$

$S_{i}^{0}$—Initial nutrient concentration in M-MPBR, i = N, P; $mg\cdot L^{-1}$;

$S_{i}$—Total nutrient concentration in M-MPBR and effluent, *i* = N, P; $mg\cdot L^{-1}$;

$\gamma_{{\omega t}_{i}}$—Nutrient consumption of microalgal metabolism, i = N, P; which

(8) $$\gamma_{{\omega t}_{i}}=-\frac{\mu_{m}X_{m}VS_{N}S_{P}}{Y_{m-i}\left(K_{N}+S_{N}\right)\left(K_{P}+S_{P}\right)}$$

where $Y_{i}$—Removal coefficient of nutrient, i = N, P; g algae∙g nutrient^−1^.

Under the steady state, the nutrient consumption (N and P) should be stable, which means the nutrient concentrations of effluent were constant ($V\frac{dS_{i}}{dt}$ = 0).

Thus,

(9) $$QS_{i}^{0}=QS_{i}+\gamma_{{\omega t}_{i}}=QS_{i}+\frac{\mu_{m}X_{m}VS_{N}S_{P}}{Y_{m-i}\left(K_{N}+S_{N}\right)\left(K_{P}+S_{P}\right)}$$

Equation (9) was divided by *Q*, and we obtained the following:

(10) $$S_{i}^{0}=S_{i}+\frac{V}{Q}X_{m}\cdot \frac{\mu_{m}S_{N}S_{P}}{Y_{m-i}\left(K_{N}+S_{N}\right)\left(K_{P}+S_{P}\right)}$$

Based on Equation (1), Equation (9) can be simplified to

(11) $$S_{i}^{0}=S_{i}+HRT\frac{{Xm\mu }_{m}S_{N}S_{P}}{Y_{m-i}\left(K_{N}+S_{N}\right)\left(K_{P}+S_{P}\right)}$$

$S_{i,i=N\;and\;P}^{0}$—Influent concentration N and P, respectively, $mg\cdot L^{-1}$;

$S_{i,i=N\;and\;P}$—Effluent concentration N and P, respectively, $mg\cdot L^{-1}$;

$Y_{m-i,i=N\;and\;P}$—Cell yield based on N removal ($Y_{m-N}$, mg cells/mg N removed) or P removal ($Y_{m-P}$, mg cells/mg P removed).

Thus, the mass balance equation for N is as follows:

(12) $$S_{N}^{0}=S_{N}+HRT\frac{{Xm\mu }_{m}S_{N}S_{P}}{Y_{m-N}\left(K_{N}+S_{N}\right)\left(K_{P}+S_{P}\right)}$$

The mass balance equation for P is as follows:

(13) $$S_{p}^{0}=S_{P}+HRT\frac{{Xm\mu }_{m}S_{N}S_{P}}{Y_{m-P}\left(K_{N}+S_{N}\right)\left(K_{P}+S_{P}\right)}$$

### 2.2. Model Variables

Biokinetic and stoichiometric parameters were employed based on the general microalgae growth kinetics and stoichiometry from previous studies [35,36]. They are listed in the following Table 1:

As a result, by changing the input conditions (HRT, SRT, and influent N concentration (S_N_^0^)), the output variables of biological performance (microalgal biomass concentration X_m_, effluent N (S_N_) and P (S_P_) concentrations in the models (Equations (6), (12), and (13)) were solved by using Microsoft Excel 2019 with a solver function. The influent P concentration (S_P_^0^) was assumed to be constant (5 mg/L) in this study to reduce the unnecessary repetition of research works.

## 3. Results

### 3.1. Modelling of Biological Performances of Membrane Photobioreactor

**Figure 2:**
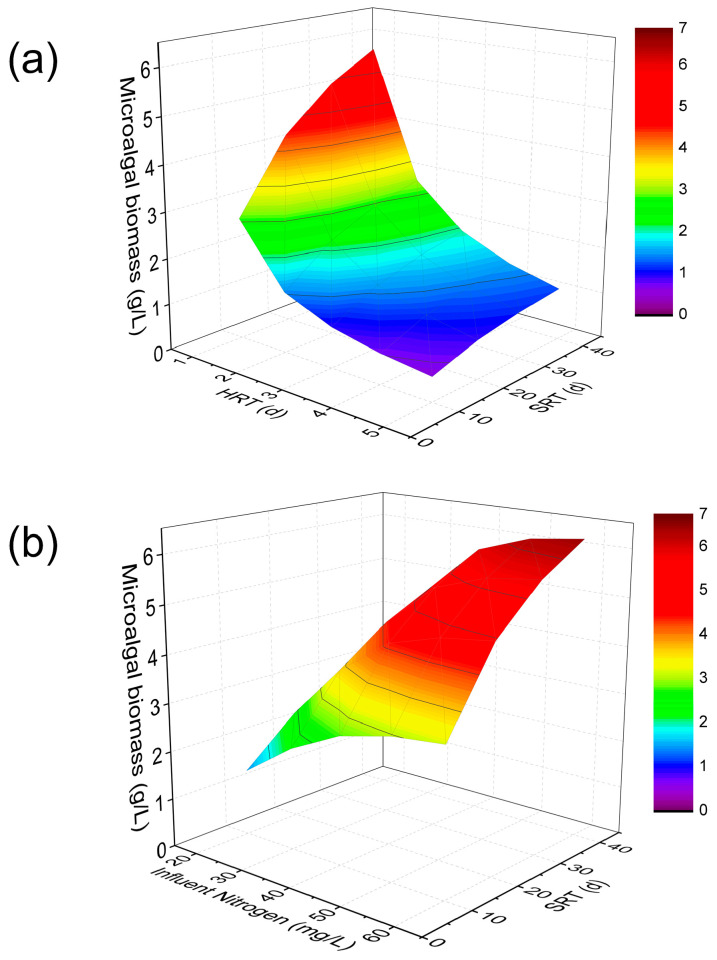
(**a**) Microalgal biomass concentration under different SRTs and HRTs at influent N = 40 mg/L and P = 5 mg/L; and (**b**) microalgal biomass concentration under different SRTs and influent nitrogen concentrations at HRT = 1 d, influent P = 5 mg/L (Single column).

**Figure 3:**
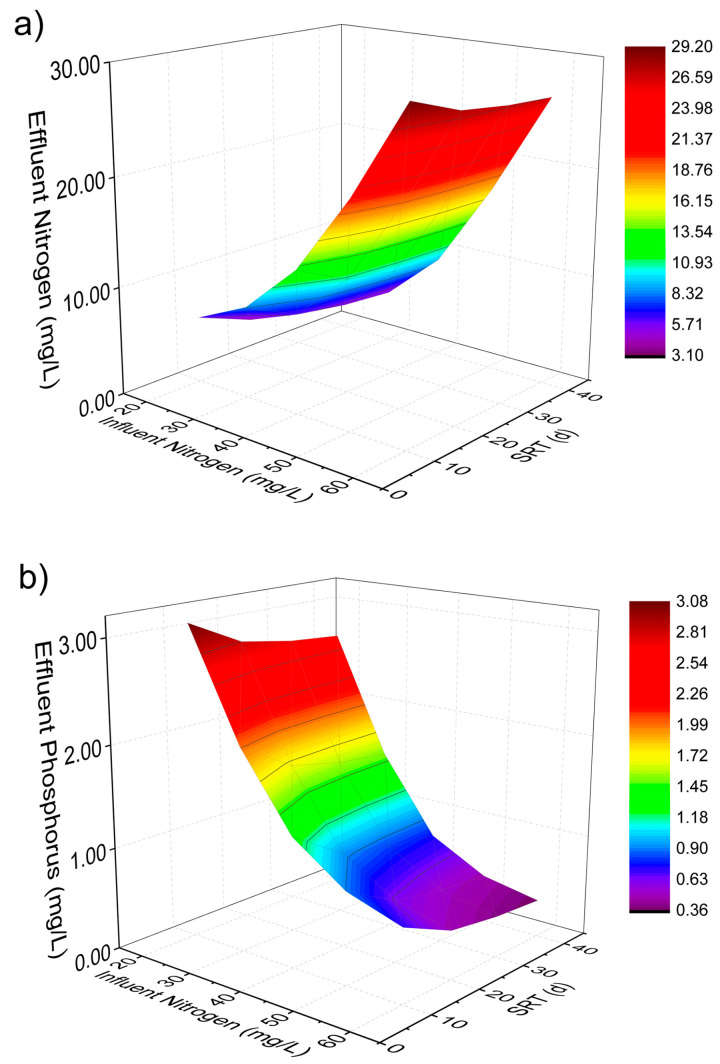
(**a**) Nitrogen and (**b**) phosphorus concentrations of effluent under different SRTs and influent N concentrations at HRT = 1 d, influent P = 5 mg/L (Single column).

**Figure 4:**
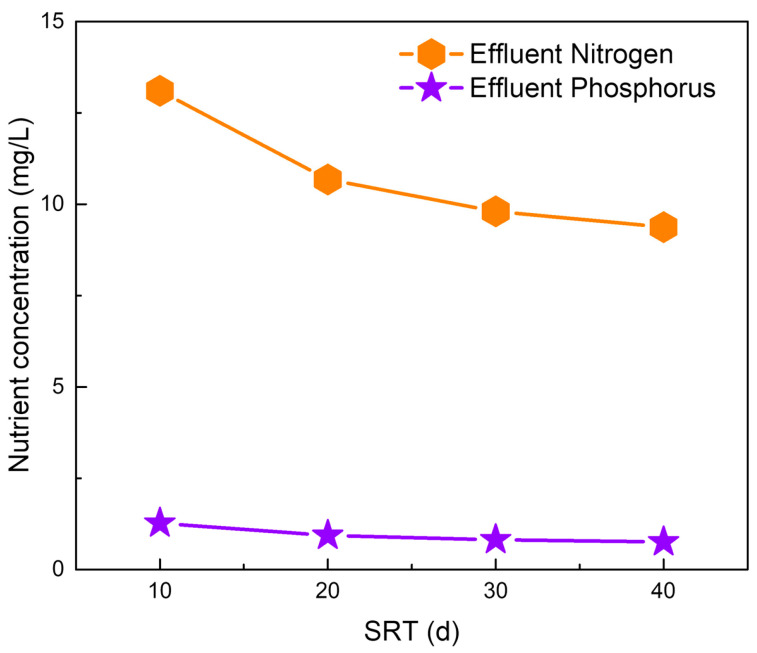
Nutrient (N and P) profiles of effluent under different SRTs at HRT = 1 d with influent N = 40 mg/L (Single column).

Figure 2 and Figure 3 show the biomass concentration profile in the M-MPBR and nutrient profiles in the effluent under different operating conditions (HRT: 1 to 5 d, SRT: 10 to 40 d, influent N concentration: 20 to 60 mg/L, and influent P = 5 mg/L). As shown in Figure 2, the microalgal biomass concentration increased with an increase in SRT and influent N concentration, while it decreased with an increase in HRT. At SRT = 40 d and HRT = 1 d, the maximum microalgal biomass concentration increased from 5.69 g/L (Figure 2a) to 6.21 g/L (Figure 2b) when the influent N concentration increased from 40 mg/L to 60 mg/L. From Figure 3, it is clear that an increase in SRT led to a decrease in both effluent N and P concentration at a fixed influent N concentration. Furthermore, an increase in influent N concentration resulted in an increase in effluent N concentration at the same SRT and influent P concentration (5 mg/L). The maximum effluent N concentration is 29 mg/L at HRT = 1 d, SRT = 10 d, and influent N concentration = 60 mg/L. The effluent P concentration increased with the decrease in the influent N concentration and SRT. The maximum effluent P concentration is 3.08 mg/L at HRT = 1 d, SRT = 10 d, and influent N concentration = 20 mg/L. The effluent N and P concentration decreased from 13.10 mg/L to 9.37 mg/L and 1.27 mg/L to 0.76 mg/L, respectively, with an increase in SRT at HRT = 1 d and influent N and P concentration = 40 mg/L and 5 mg/L, respectively (Figure 4).

The general trends of changes in effluent N and P concentrations under different SRTs, HRTs, and influent N and P concentrations are consistent with the ones predicted by microbial kinetic models and mass balance equations for conventional continuous stirred tank photobioreactors [37]. This finding suggests that the predictions of these parameters are reasonable, based on the mathematical models established here for the M-MPBR system.

### 3.2. Model Validation of Microalgae System

**Figure 5:**
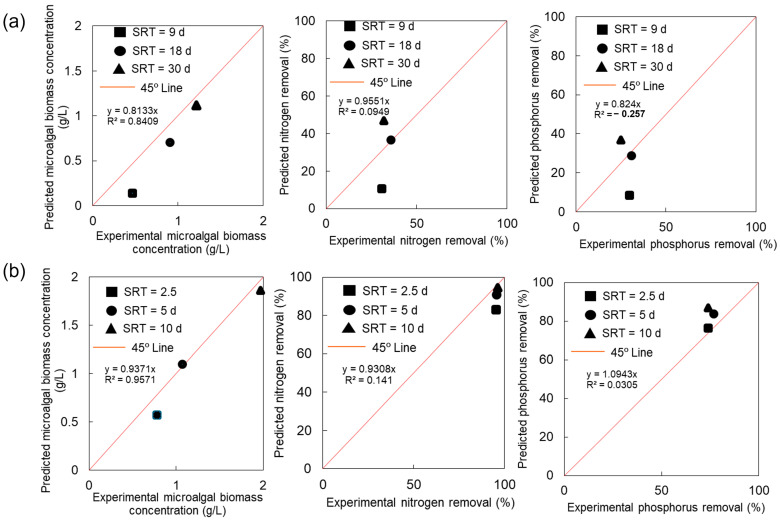
Model validation with experimental results of (**a**) Luo et al.’s work [19] and (**b**) Praveen et al.’s work [38]. (Double column).

The accuracy of model predictions was validated with the experimental results from the literature [19,38]. The operating conditions and biological performances of M-MPBR systems in the literature [19,38] are summarized in Table 2. The experimental results show that an increase in SRT led to an increase in the microalgal biomass concentration, but the nitrogen (N) and phosphorus (P) removals did not change much under tested SRTs and conditions. The biokinetic parameters and stoichiometric coefficients (Table 3) used to predict the biological performance (microalgal biomass concentration, nitrogen (N), and phosphorus (P) removal efficiencies) were from the literature and/or calibrated, based on the experimental results [19,38]. Figure 5 shows a good linear correlation (Figure 5a (coefficient of determination R^2^ = 0.8405) and Figure 5b (R^2^ = 0.9571)) of microalgal biomass concentration between the predicted and experimental results from the literature. However, there are significant linear correlations of either nitrogen (N) or phosphorus (P) between the predicted and experimental results at different SRTs from the literature (Figure 5a (R^2^ = −0.257–0.0949) and Figure 5b (R^2^ = 0.0305–0.141)). The data points on the 45° line suggest a perfect match between the prediction and experimental results. Almost all the predicted and experimental points exhibited minor deviations to a 45° line, while a little bit larger deviations were found on nutrient (N and P) removals in the Luo et al. [19] case. The modelling results are generally in good agreement with the experimental results from the literature, considering the fact that the biokinetic and stoichiometric parameters used in this study (Table 3) are from the literature [35,36] with no or minimal calibration for the specific microalgae species and systems used in these experiments.

## 4. Discussion

### 4.1. HRT Effect

As reported in the literature, HRT is an important process parameter of M-MPBR operations, affecting pollutant removals and microalgae production [14,19,28]. In general, a shorter HRT corresponds to a higher nutrient loading rate, which could provide sufficient nutrients for microalgae growth in the bioreactor, especially in low-medium strength wastewater cases [28]. Some studies reported that microalgal biomass production could be promoted by longer HRTs because it prolongs the contact between microalgae and suspended nutrients, thus increasing the metabolism period [39]. Lee et al. [39] found that the *Chlorella vulgaris* biomass concentration decreased from 0.8 g/L to 0.4 g/L when the HRT decreased from 6 days to 3 days. This reduction in biomass production was ascribed to the high loading rate of toxic chromium (VI) under the shorter HRT (3 d), which was out of the limitation of microalgae degradation, thus causing the decay of microalgae [39]. There is also an argument that a longer HRT contributes to the reduction in microalgal biomass due to the lower nutrient loading and higher decay rate caused by the nutrient shortage [40]. Ashadullah et al. [27] found that a longer HRT exhibited a better nutrient removal performance. The total N removal increased from 61.3% to 67.5% when the HRT increased from 2 days to 7 days [27]. However, the nutrient profiles in this study have not shown a very obvious correlative changing trend with HRT changing, compared to the experimental case. The reason might be that nutrient removal involves multiple mechanisms (microalgae assimilation, ammonia volatilization), and nutrients in suspended solids need a long time for hydrolysis, which is also influenced by HRT except for biomass production [41]. Differently, the mono-microalgal model from this study only considered the soluble nutrients (N and P) consumed by biomass production.

Despite the worldwide acceptance that a longer HRT improves biodegradation, photodegradation, and sorption, particularly for non-soluble CODs and nutrients (N and P), leading to higher consumption of pollutants [42], it does not mean that the photobioreactor would benefit from a longer HRT condition if the biodegradation rate is fast enough. The lower organic loading rate traced back to a longer HRT restricted the microalgal biomass production even though a longer HRT provides a longer biodegradation period to improve the effluent quality [40,43]. At this point, the long HRT and low biomass production are not applicable for industrial applications due to the high energy and capital costs.

### 4.2. SRT Effect

Solid retention time (SRT) is a key design and operation parameter of the M-MPBR that determines the biological performance of the M-MPBR. The literature claims that a longer SRT could enhance nutrient recovery and then facilitate microalgae accumulation, increasing biomass production [20,44,45]. As a result, the microalgal biomass concentration increased with the increase in SRT (Figure 3), implying that the trend of mathematical modelling of microalgae biomass concentration changes with respect to SRT is correct. Wang et al. [46] also reported that the total N and P concentrations in the effluent of an osmotic photobioreactor decreased when the SRT increased from 9.41 d to 25.26 d. The trend of the modelling results of nutrient removals (Figure 3) is consistent with that of the experimental results from the literature [46]. However, the upper limit of SRT for the purpose of increased microalgal biomass concentration to increase nutrient (N and P) removals is limited by the light self-shading effect at a higher microalgal biomass concentration [32,47]. Thus, other factors, such as light intensity and gaseous CO_2_ concentration limitations should be considered in future models.

Nutrient removal could also be affected by the SRT. Previous studies have found a linear correlation between microalgal biomass production and nutrient (N and P) uptake in either batch studies or photobioreactor studies [48,49]. Briefly, a longer SRT contributes to a higher microalgal biomass concentration due to its lower wasting rate and longer harvest interval [50]. Xu et al. [21] reported that the microalgal biomass concentration increased from 0.69 g/L to 1.47 g/L when the SRT rose from 5 days to 10 days. Ignoring the limitation of other process parameters (such as light and carbon dioxide), a higher SRT indeed results in a higher microalgal biomass concentration, which usually comes with a higher nutrient removal due to the larger nutrient consumption caused by more microalgal biomass. It appears that a longer SRT might be more acceptable, due to the higher biomass concentration and higher nutrient removal. However, the positive effect brought by SRT could be restricted by other factors in real situations, such as the limitation of light penetration distance in a concentrated microalgae suspension. As Discart et al. [51] mentioned, the light attenuation and carbon dioxide shortage would happen when the biomass concentration reaches a high level that will cause higher decay efficiency, thus decreasing the biomass productivity. As a result, a poor N removal could be obtained because the majority of N removal relied on algal biomass growth and wasting [21]. Some experimental studies also observed similar behaviours that the N removal decreased with the increase in SRT [52,53]. The modelling results from this study did not show similar trends under the tested SRT range (10–40 d). This finding could be attributed to the fact that the range of SRT (10–40 d) tested here was not long enough that microalgae decay became dominant. It can also be attributed to the fact that the majority of the N removal was carried out by the microalgae uptake, and there was no significant release of proteins from the microalgae decay, which only correlated to the microalgal biomass.

### 4.3. Influent Nitrogen Concentration Effect

It has been reported in the literature that the ratio of nutrients (N/P) in the influent has an effect on the biological performance of an M-MPBR, particularly on microalgal biomass production [14]. As shown in Figure 2, the microalgal biomass concentration increased when the nitrogen concentration of influent increased from 20 mg/L to 60 mg/L at HRT = 1 d and SRT = 40 d. As a key process parameter of microalgae growth, the augment of influent N concentration has a promoting effect on microalgae productivity [54]. When the HRT and SRT are constants, and light and gaseous CO_2_ concentration are not the limiting factors, the wastewater characteristics, such as influent N concentration, are the only factors affecting microalgal biomass concentration. As shown in this model (Equation (11)), the microalgal biomass concentration on the right side of nutrient mass balance equations increased with the rising nutrient concentration of influent to maintain the mass balance at a steady state. When it came to the nutrient profile, the situation became a little bit of more complex. The effluent N concentration increased with an increase in influent N concentration, while the effluent P concentration decreased (Figure 3). This phenomenon could be attributed to the effect of nutrient limitation [55]. At a low influent N concentration, insufficient nitrogen content in the influent could not support microalgae to consume all remaining P in the influent. As a result, the M-MPBR had a high N removal (84.1%) but low P removal (46.7%) efficiency. Moreover, when the influent N concentration increased, more N could be utilized to support the P uptake. Thus, the P removal increased with an increase in the influent N concentration. However, the P content would become the limitation that restricts N consumption, if a high level of influent N concentration is further increased. Thereby, the M-MPBR system had a low N removal (55.8%) but high P removal (92.7%). It appears that there is an optimal N/P ratio that would benefit microalgae growth and achieve the discharge standard of both effluent N and P after treatment. Wang et al. [56] also claimed that an extremely high or low N/P ratio of influent contributed to a decline in microalgae growth. Their study reported that the optimal N/P ratio for algal growth was around 6.8–10, which also agrees with the modelling prediction from this study (Figure 2 and Figure 3). Moreover, this N/P ratio is also close to the ratio in domestic wastewater and secondary effluent (7.5–9.6) [57], which indicates that the M-MPBR has a high potential in domestic wastewater treatment applications.

In summary, suitable operating conditions can improve the biological performance of the M-MPBR system. Based on the established models and used biokinetic and stoichiometric parameter values, an influent N concentration = 40 mg/L, P concentration = 5 mg/L, HRT = 1 day, and SRT = 40 days appears to be the optimal operating conditions for a high biomass production and high nutrient removal (both N and P). The mathematical models proposed in this study can predict changes in the biological performance of the M-MPBR system under different process conditions (SRT and HRT) and wastewater characteristics (N/P ratio).

### 4.4. Comparison of the Modelling and Experimental Results

The prediction and validation results described in the previous sections suggest that the mathematical models developed from this study can reasonably predict the impacts of process variables and wastewater characteristics on the biological performance of the M-MPBR system. Most of the data points (Figure 5) are on or close to the 45° line (except nutrient (N and P) removal in the Luo et al. [19] case). A small deviation of the 45° line suggests the predicted results are in excellent or very good agreement with the experimental results. The accuracy of model prediction can be further improved, if more suitable biokinetic parameters and stoichiometric coefficients are used for these specific (species) experimental systems. The biokinetic parameters and stoichiometric coefficients used in this study are the typical values for microalgae but may not be perfect for the microalgae species used in the literature [19,36]. A calibration of the biokinetic parameters and stoichiometric coefficients for specific microalgae species would improve the accuracy of the mathematical prediction developed in this study for the M-MPBR system using the specific microalgae species for nutrient (N and P) uptakes and biomass cultivation.

There are relatively larger deviations of the nutrient (N and P) removal efficiencies between the modelling results and the experimental results from Luo et al. [19], while the validation shows that a good fit between the experimental and modelling data was achieved for the experimental work of Praveen et al. [36]. The biomass concentration from modelling was slightly lower than that of the experimental results of Luo et al. [19]. This finding could be attributed to the fact that some bacteria existed in the microalgal biomass, which was not considered in the models (only for the mono-species of microalgae but not the co-existence of bacteria). The improved and higher nutrient (N and P) removal efficiencies in shorter SRT from Luo et al. [19], as compared to the modelling results, could be attributed to the contribution of the additional bacterial nutrient (N and P) uptake, which was not considered in the modelling study, at SRT = 9 d; on the other hand, the deteriorated and decreased nutrient (N and P) removal efficiencies of Luo et al. [19] at SRT = 30 d, as compared to the modelling results, might be attributed to the light penetration limitation at a higher microalgal biomass concentration at SRT = 30 d. The modelling results have better agreements with the experimental results of Praveen et al. [36], as compared to that of Luo et al. [19] (data points from Praveen et al. [36] closer to the 45° line). This finding might suggest that the used values of the biokinetic parameters and stoichiometric constants are more suitable for the species and conditions of Praveen et al. [36]. It is anticipated that a better agreement between modelling and experimental results can be anticipated if the values of the biokinetic parameters and stoichiometric constants are calibrated for the specific species and experimental conditions.

The model validation indicated that the real situation is more complicated than the mathematic models can predict, as assumptions are made in deriving these models. Unlike this model in which phosphorus was only consumed by microorganisms, the nutrient removal routes are diverse (biological and physicochemical) and would happen together simultaneously to affect the nutrient profile, thus making it a challenge to predict [58,59]. Moreover, the mono-microalgae system in wastewater would face the risk of bacteria contamination, which could impact biomass production and nutrient removal [60]. Therefore, more considerations such as interaction with bacteria should be taken into account in future model development.

## 5. Conclusions

A set of mathematical models was, for the first time, developed for the prediction of the biological performance of the M-MPBR system. The developed mathematical models can effectively predict the impacts of process variables (SRT and HRT) and wastewater characteristics on the biological performance (microalgal biomass production, nitrogen and phosphorus removals) of the M-MPBR system. The modelling results suggest that there is an optimal SRT and N/P ratio for enhanced microalgal biomass production and N and P removal. The modelling results are in good agreement with experimental results from the literature. For the mono-microalgal-based system, biomass production and nutrient removal increased with an increase in SRT and a decrease in HRT. The biomass production increased with an increase in the influent N concentration, while nutrient (N and P) removal could be restricted under extreme conditions (either too high or too low N concentration of the influent). The optimal conditions (HRT = 1 day, SRT = 40 days, and the influent of nitrogen concentration = 40 mg/L) existed for enhanced high biomass production and acceptable nutrient (N and P) removal. The developed mathematical models can be used as a powerful tool for optimal design and the operation of the M-MPBR system for sustainable wastewater treatment. However, microalgae cultivation in real nature is influenced by a number of factors, such as competition from other species (either other microalgal or even bacterial species), CO_2_ concentration, temperature, pH, and light intensity besides HRT, SRT, and wastewater characteristics. A more complicated model that includes the impacts of other factors should be considered in future studies.

## Figures and Tables

**Table 1:** Growth biokinetic and stoichiometric coefficients for a modelling study (Figure 1, Figure 2, Figure 3 and Figure 4).

Kinetic Parameters	Value	Ref
µ_m_ (d^−1^)	1.68	[35,36]
k_d-m_ (d^−1^)	0.06	[35,36]
Y_M-N_ (mg biomass·mg N^−1^)	15.8	[35,36]
Y_M-P_ (mg biomass·mg P^−1^)	114	[35,36]
K_N_ (mg N·L^−1^)	24.5	[35,36]
K_P_ (mg P·L^−1^)	3.39	[35,36]

**Table 2:** Operation conditions and biological performance of the M-MPBR systems for validation of the models.

	SRT (d)	HRT (d)	Influent Nitrogen (mg/L)	Influent Phosphorus (mg/L)	Average Biomass Production (g/L)	Average Nitrogen Removal (%)	Average Phosphorus Removal (%)
Luo et al. [19]							
	9	1	14.10	2.50	0.47	31.00	30.00
	18	1	14.10	2.50	0.91	36.00	31.00
	30	1	14.10	2.50	1.22	32.00	25.00
Praveen et al. [38]							
	2.5	2	21.00	6.00	0.78	95.5	78.2
	5	2	21.00	6.00	1.07	95.8	74.1
	10	2	21.00	6.00	1.97	96.7	73.1

**Table 3:** Growth biokinetic and stoichiometric parameters for validation of the accuracy of the models.

Kinetic Parameters	Value		Ref
	Luo et al. [19]	Praveen et al. [38]	
µ_m_ (d^−1^)	1.68	2.45	[35,36]
k_d-m_ (d^−1^)	0.06	0.06	[35,36]
Y_M−N_ (mg biomass·mg N^−1^)	15.8	30 *	[35,36]
Y_M−P_ (mg biomass·mg P^−1^)	114	114	[35,36]
K_N_ (mg N·L^−1^)	24.5	12.1	[35,36]
K_P_ (mg P·L^−1^)	3.39	0.27	[35,36]

* Calibrated.

## Data Availability

The data presented in this study are available upon request from the corresponding author.

## Abbreviations

**Symbol**	**Description**	**Unit**
M-MPBR	Microalgal membrane photobioreactor	
M_Input_	The mass input to the system	
M_Output_	The mass output from the system	
M_Accumulation_	The mass accumulated in the system	
N	Nitrogen	
P	Phosphorus	
HRT	Hydraulic retention time	d
SRT	Solid retention time	d
V	Effective volume of microalgae M-MPBR	L
Q	Flow rate of influent	L∙d^−1^
Q^W^	Waste rate of microalgae suspension	L∙d^−1^
X_m_	Microalgal biomass concentration	mg∙L^−1^
X_m_^0^	The initial concentration of microalgae in M-MPBR	mg∙L^−1^
µ_m_	Maximum growth rate of microalgae	d^−1^
k_d-m_	The decay coefficient of microalgae	d^−1^
S_N_	Total nitrogen concentration in M-MPBR and effluent	mg∙L^−1^
S_P_	Total phosphorus concentration in M-MPBR and effluent	mg∙L^−1^
K_N_	The half-saturation constant of NH_4_^+^-N	mg N∙L^−1^
K_P_	The half-saturation constant of HPO_4_^2−^-P	mg P∙L^−1^
S_i_^0^	Initial nutrient concentration in M-MPBR, i = N, P	mg∙L^−1^
$\gamma_{{\omega t}_{i}}$	Nutrient consumption of microalgal metabolism, i = N, P	
Y_m−i_	Removal coefficient of nutrient, i = N, P	g algae∙g nutrient^−1^
